# Do the Sick Fear Death?

**Published:** 1890-05-10

**Authors:** 


### Do the Sick Fear Death?

"Close up his eyes, and draw the curtains close, and let us all to meditation." Let us meditate on Death—that word, which even to pronounce many people are averse, preferring the paraphrase, "All over." For there is an innate shrinking of dust with the breath of life in it from dust out of which the breath of life has departed. When the doctor leaves the house, and the blinds are drawn down, there are usually and fortunately duties to be performed which in some degree distract attention from the loss. Relatives and friends must be informed, funeral arrangements made, the garments of woe demanded by society obtained, and the provision of funeral baked meats, which is not yet gone quite out of fashion, must be attended to. It is well that it should be so, for occupation of the mind is the great antidote to grief. When clay has been returned to clay, and the decorous cheerfulness usual on such occasions is regaining ascendancy, we begin to speak of a "happy release," God's light again brightens the house, and we drift back as nearly as possible to the old groove. We soon forget the warning that has been given us, and we rarely apply the lesson to ourselves, for "All men think all men mortal but themselves." The query, "What is death?" will be replied to differently, according to the belief of the individual essaying an answer. The Christian will say it is the separation of the soul or vital principle from the body. It is true that we do not know what the soul is, or what the vital principle is. It has been argued that they are distinct. This, however, we do know, viz., that if the vital principle or life departs, the soul does not linger. It is, therefore, only consistent with reason to presume that, if not identical, they are incapable of separate existence in the human body. Life, it is true, may remain without mind or intellect being manifest, as, for instance, after an injury of the brain. But the mind must still be there, although incapable of asserting itself, for conditions of insensibility lasting days, hours, weeks, even months and years have been recovered from. The advanced agnostic, however, would give a different reply to the question, "What is death?" and say it is the extinction of the vital principle, also regarding mind as not of divine origin, but as the product of evolution and education. But can the inconceivable essence we call life ever become extinct; does it not rather return to the infinite? The extinction of life has been compared with the abolition of light when a candle is blown out. But we do not know sufficient of the subject to venture on any such comparison. It belongs to the series of problems which have vexed mankind from ancient times till the present day. We are no more able to assert anything regarding the matter than Protagoras was when condemned to death by the Athenians, because he confessed himself unable to say whether or not the gods existed.

We therefore turn to another point which is more susceptible of explanation. Sir Lyon Playfair has recently asked various medical men if their patients feared death? With several exceptions, including Sir William Gull and Sir Robert Christison, medical men have answered, "No!" This is scarcely in accordance with our experience, which leads us to assert that a great many sick people do fear to die. Much, however, depends on the manner in which people die, or, in other words, on the mechanism of death. When a person is rendered insensible by a blow on the head, or by the rupture of a vessel in the brain, and does not recover from such insensibility, there can be no fear of death. For fear, being a mental emotion, implies the possession of the mental faculties and the action of the mind. Again, when a man is in the jaws of a wild beast, we are credibly informed by persons who have been so seized, that there was no fear of death, often even no pain felt. The energies are engaged in the encounter and in the struggle for dear life, and fear does not gain the ascendency. The feeling as described by persons in this peril is rather one of exasperation against the animal they are struggling with. When actually engaged in battles soldiers rarely experience fear, whatever may be the mental state previously. The excitement, noise, numbers, and the desire to kill aroused dispel fear. It is the same in a sinking ship, where persons hurrying to save their lives have no time to think of death. Again, neither infants nor very old people fear death. Infants cannot do so, as their mental faculties are not sufficiently developed. Old people do not do so when they arrive at the condition "sans eyes, sans teeth, sans everything," or even when the mental faculties are on the wane.

Experience of death-beds shows that all persons do not die in the same manner. Death beginning at the brain has already been referred to when mentioning insensibility produced by a blow on the head. But death may occur from failure of the circulation, or it may occur from some obstruction to the entrance of air into the lungs. In such cases death may sometimes be sudden; but it is often very lingering, and the mental faculties may be retained till the last. When this is the case there are various reasons causing fear of death. There may be fear of the actual pain of dying—of what is well termed the "agony of death." It has been argued that death is painless, and that the so-termed "agony" is really caused by the struggle to live. This may be scientifically and physiologically correct. But it does not much signify to the dying how the pain is caused. The pain is there at the time of dying, and it is the pain which is feared. Of course we do not exactly know what pain persons experience when dying, for no one returns from the other world to tell us. But that pain is often experienced at the hour of death is beyond question, and as a long dream occupies but a few moments, so much agony may be compressed into the hour of death.

Bacon said men fear death as children fear the dark. There is the uncertainty of the afterwards. Whether there is an afterwards is doubted by some. Those who think the soul is extinct when it leaves the body do not, of course, believe in afterwards. But notwithstanding this, they often *doubt*, and, like Voltaire, are liable to recant when near the necessary end. Should they, however, be firmly impressed that there is no afterwards, they can only feel fear of death in consequence of the pain attending it.

Again, fear of death may be caused by a conviction of damnation. When, after an ill-spent life, the hour of dissolution approaches, if there is a belief in the pit of torment, and no hope of pardon, the last hours may be of terror and horror.

On the other hand, when a person has the assurance and vanity to believe that he has lived his life better than his neighbours, or that on other and better grounds he is "saved;" that after death he will be received direct to Abraham's bosom, there will be no fear of death except from actual pain.

A great deal depends on what persons can believe. The fanatical Mahommedan does not fear death, even when hanged for killing a Christian, for he has the firm belief that the crime is his passport to heaven. The Eastern ascetic, who has approached to the attainment of *nirvana*, does not fear death. As a matter of fact, the teaching of any religion may suffice to dispel fear of death. The Christian religion, perhaps, above all others, is calculated not only to bring consolation to, and prevent fear of, death, but also to implant a fear of it.

It has been argued that men would not commit suicide if they feared death. But the question has never been satisfactorily answered, whether or not a suicide was in the possession of his right senses. It is well known that many insane persons have the suicidal tendency. It has been advanced, that the fact of suicide implies some brain defect. The verdict of a coroner's jury on a suicide is almost invariably "temporary insanity." And we know that there is such a condition as hereditary suicidal tendency. When we recollect the very trivial causes which induce people to commit suicide it certainly does appear that there must be some brain defect.

Our conclusions are, therefore, that a great many persons, perhaps the majority, do fear death, but that the fear arises from various causes, and is distinctly related to the belief entertained, and to the manner, mode, or mechanism of the death. If we could all think with Goethe, that immortality is not an inheritance, but a greatness to be achieved like any other greatness by courage, self-denial, and purity of purpose, there would be less fear of death than there now is. But we suppose this would tend too much towards Agnosticism to be well received by the Church, which confers immortality on all human beings. On this head we suggest a perusal of Laing's "Problems of the Future."

